# Structural basis of cuproenzyme nitrite reduction at the level of a single hydrogen atom

**DOI:** 10.1016/j.jbc.2025.110290

**Published:** 2025-05-26

**Authors:** Yohta Fukuda, Masami Lintuluoto, Yu Hirano, Katsuhiro Kusaka, Tsuyoshi Inoue, Taro Tamada

**Affiliations:** 1 Graduate School of Pharmaceutical Science, Osaka University, Suita, Osaka, Japan; 2 Integrated Frontier Research for Medical Science Division, Institute for Open and Transdisciplinary Research Initiatives (OTRI), Osaka University, Suita, Osaka, Japan; 3 Graduate School of Life and Environmental Sciences, Kyoto Prefectural University, Kyoto, Japan; 4 Institute for Quantum Life Science, National Institutes for Quantum Science and Technology, Chiba, Chiba, Japan; 5 Center of Quantum Life Science for Structural Therapeutics, Chiba University, Chiba, Chiba, Japan; 6 Neutron Industrial Application Promotion Center, Comprehensive Research Organization for Science and Society, Tokai, Ibaraki, Japan; 7 Center for Infectious Disease Education and Research (CiDER), Osaka University, Suita, Osaka, Japan

**Keywords:** neutron crystallography, copper-containing nitrite reductase, structural biology

## Abstract

Hydrogen (H) atoms account for about half the atoms in biomacromolecules and are essential for their biochemical properties such as enzymatic functions. Obtaining precise enzyme structures that include all the H atoms allows a deeper understanding of their structure-function relationships. Copper-containing nitrite reductases (CuNIRs) catalyze transformation of nitrite to nitric oxide, which has impacts on geochemical, agricultural, and medical health fields. Despite intense research efforts, the dynamics of H atoms during the enzymatic reaction of CuNIRs are unknown and hence the catalytic mechanism remains unclear. We performed neutron crystallography to shoot a single H-atom resolution picture of a CuNIR in complex with nitrite. We found that nitrite binds on the catalytic Cu center as nitrite (NO_2_^-^) and not as protonated HNO_2_. Our X-ray data and quantum chemical calculation show that NO_2_^-^ is in an electron-localized state that can facilitate N-O bond cleavage after receiving an electron. The catalytic residues, Asp^CAT^ and His^CAT^, are deprotonated and protonated, respectively, suggesting that His^CAT^ is the point of departure of the proton transfer sequence. Quantum chemical calculations show that the neutron structure is consistent with the Cu(II) state and that the highly polarized state of the catalytic site is stabilized by the permittivity of solvent molecules filling a water channel. Subatomic resolution X-ray structures of the Asp^CAT^-to-Asn mutants, which mimic the protonated state of Asp^CAT^, were also determined to investigate the involvement of protonated Asp^CAT^ in the reaction. Our crystallographic data and quantum chemical calculations reveal in detail the first step of the CuNIR reaction.

The one electron reduction reaction of nitrite (NO_2_^-^) to nitric oxide (NO) occupies a crucial position in the global geobiochemical system. It contributes to a key step of the denitrification process which converts nitrogen oxides in soil and water to gaseous dinitrogen (N_2_) that is subsequently released to the atmosphere (1). NO_2_^-^ reduction in denitrification is important agriculturally because it irreversibly removes nitrogenous fertilizer from soils and thus impacts crop yields (2, 3). Furthermore, clinically important pathogens also possess denitrification genes, allowing the production of NO from NO_2_^-^ to adapt to environmental changes; therefore, NO-forming enzymes are potential drug targets (4, 5, 6, 7).

The NO generation from NO_2_^-^ in the denitrification process is catalyzed by dissimilatory NO_2_^-^ reductases containing iron or copper cofactors at their catalytic centers (8). Organisms having such enzymes are found in almost all environments on Earth. Copper-containing nitrite reductases (CuNIRs) are particularly ubiquitous and found in all three domains of life, bacteria, archaea, and eukaryota (1, 9, 10, 11). CuNIRs typically have an ∼110 kDa homotrimeric structure, like a Reuleaux triangle (Fig. 1*A*). Each protomer contains one type 1 copper (T1Cu) and one type 2 copper (T2Cu) site (12, 13). The T1Cu site, also known as the blue copper site due to its blueish color in the oxidized Cu(II) state, accepts an electron from physiological electron donors. The T1Cu sites in CuNIRs are usually coordinated by one methionine (Met), one cysteine (Cys), and two histidine (His) residues. The Cys residue forms a peptide-bond bridge (Cys-His bridge) together with one of the His ligands at the T2Cu site to transfer the electron to the catalytic T2Cu site, which is coordinated by three His residues and an axial water molecule. The axial water is located at the end of a water/proton channel that leads to the molecule’s surface. Experimental (14) and computational (15) studies support that the intramolecular electron transfer (ET) pathway through the Cys-His bridge involves an H-bond jump between the Cys amide O atom and the imidazole N^δ^ atom of the T2Cu ligand His. Two conserved catalytic residues, aspartate (Asp^CAT^) and His (His^CAT^), located above the T2Cu site, both assist in proton transfer (PT) to the substrate (16, 17). These residues are connected *via* one water molecule called bridging water (BW).

**Figure 1:**
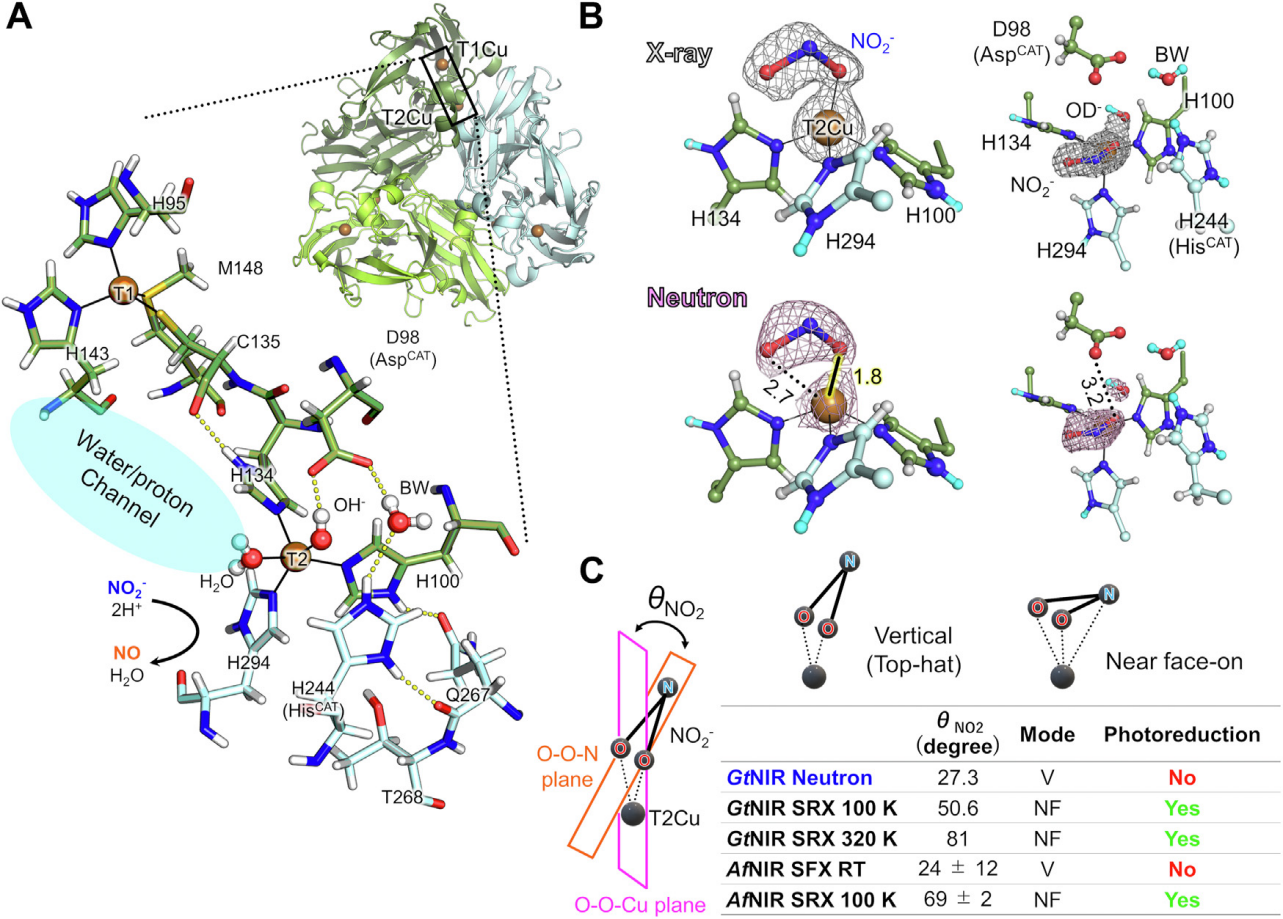
**Neutron structure of *Gt*NIR.***A*, the overall structure of *Gt*NIR (PDB code: 6L46; neutron structure of the resting state) and its copper centers. *Black lines* show coordination bonds. *Dashed yellow lines* are hydrogen bonds. *B*, the T2Cu site of the NX joint refined structure of *Gt*NIR in complex with nitrite. Sigma-A–weighted 2*F*_o_–*F*_c_ maps for X-ray (*gray*, contoured at 1.2σ) and neutron (*pink*, contoured at 1.2σ) data. *Black solid lines* indicate coordination bonds. Distances are shown in Å unit. H and D atoms are colored by *white* and *cyan*, respectively. A low-occupancy water molecule at the nitrite position has been eliminated for clarity. *C*, nitrite binding modes observed in CuNIR structures determined by various methods and at various temperatures. SRX and SFX are abbreviations for synchrotron radiation and serial femtosecond crystallography, respectively, and V and NF for vertical and near-face-on modes, respectively. CuNIR, copper-containing nitrite reductase; *Gt*NIR, CuNIR from *Geobacillus thermodenitrificans*; T2Cu, type 2 copper.

Despite intense research efforts, the detailed catalytic mechanism of CuNIR remains unclear. Of the chemical reaction, PT, which is coupled with intramolecular ET (18, 19), is the most ambiguous step because of the difficulty of directly observing H atoms at the catalytic site. We previously performed neutron diffraction (ND) crystallography on a CuNIR and revealed protonation states of the catalytic site in the resting state (14); however, observation of the H atoms in the transient and fragile enzyme–substrate complex is more challenging.

We here performed ND crystallography to obtain a structure of a CuNIR in complex with NO_2_^-^. The obtained structure was analyzed by quantum chemical calculations. Moreover, we conducted atomic resolution X-ray diffraction (XRD) crystallography of mutant enzymes in which Asp^CAT^ was replaced with Asn to mimic the unstable protonated state of Asp^CAT^.

## Results

### Cryogenic neutron structure of the NO_2_^-^-bound state

As with our previous ND crystallographic study, we used a CuNIR from *Geobacillus thermodenitrificans* (*Gt*NIR) (14). Although synchrotron X-ray data is necessary for neutron X-ray (NX) joint refinement to build a high-quality model (20), the T1Cu center is rapidly reduced by X-ray irradiation, reducing NO_2_^-^ at the T2Cu site (21, 22). We therefore used the C135A mutant, in which the Cys residue of the Cys-His bridge is replaced with Ala. The Ala residue does not coordinate to the T1Cu atom, thereby greatly suppressing ET from T1Cu to T2Cu (23, 24). Moreover, we performed the experiment at cryogenic temperature (100 K) to trap the NO_2_^-^-bound state, which spontaneously decomposes during room temperature (RT) neutron data collection over a week. We collected neutron and X-ray data from the same single crystal. NX joint refinement was performed using reflections up to 1.70 Å resolution for ND and 1.30 Å resolution for XRD data (Table S1).

The electron and the neutron-scattering length density maps clearly show the presence of an axial ligand on T2Cu (Fig. 1*B*). The ligand was modeled as a mixture of a major NO_2_^-^ ligand (occupancy 0.7) and a minor water ligand (occupancy 0.3) (Fig. S1*A*). The occupancy values were estimated based on occupancy refinement of *phenix.refine* (the same shall apply hereinafter). Calculations using other occupancy values showed residual densities (Fig. S1*B*). A hydroxide anion (a deuterated hydroxide dute [OD] ion in the deuterated environment) with an occupancy of 0.25 was also positioned above the T2Cu site as was found in the resting state neutron structure (14). The neutron-scattering length density map shows that the nitrite ligand is in the deprotonated form (NO_2_^-^) rather than in the protonated HNO_2_ form; thus, it does not form an H-bond with Asp^CAT^. The shortest distance between NO_2_^-^ and Asp^CAT^ is 3.2 Å, indicating that there is only a van der Waals contact. An FTIR spectroscopic study of a CuNIR from *Alcaligenes faecalis* (*Af*NIR) reports that carbon monoxide coordinated to the T2Cu center does not form an H-bond with Asp^CAT^ (25), which is in agreement with our result.

The NO_2_^-^ ligand shows a κ^1^-O coordination manner with Cu-O^nitrite^ distances of 1.8 and 2.7 Å (Fig. 1*B*). The κ^1^-O binding mode of NO_2_^-^ was initially modeled based on an extended X-ray absorption fine structure experiment of a CuNIR from *Achromobacter xylosoxidans* (26), but subsequent crystallographic analyses have revealed that NO_2_^-^ often shows an asymmetric κ^2^-O,O bidentate mode (13, 27, 28). One of the exceptions is a previously determined structure of *Gt*NIR in complex with NO_2_^-^ (24). It shows NO_2_^-^ in a κ^1^-O form with highly asymmetric Cu-O^nitrite^ distances of 2.0 and 3.4 Å. Compared to it, the NO_2_^-^ ligand in our neutron structure is closer to the κ^2^-O,O bidentate manner (Fig. S2), and similar binding modes have been reported as bidentate coordination. For example, the NO_2_^-^ ligand in a CuNIR from *Neisseria gonorrhoeae* shows Cu-O^nitrite^ distances of 2.00 ± 0.11 and 2.56 ± 0.12 Å (29), similar to the bond distances observed in our neutron structure.

The angle (*θ*_NO2_) between the plane defined by T2Cu and the two O atoms of NO_2_^-^ and the plane defined by the three NO_2_^-^ atoms is 27.3° in the neutron structure, compared to 50.6° in the previous X-ray structure (Fig. 1*C*). The coordination geometry of NO_2_^-^ in the neutron structure is the vertical mode that binds to the T2Cu atom only through O atoms, and not the near-face-on mode in which all three atoms of NO_2_^-^ are close to the T2Cu atom. The vertical mode was observed in a damage-free structure of *Af*NIR (*θ*_NO2_ = 24 ± 12°) determined by serial femtosecond crystallography, whereas the near-face-on mode is often found in photoreduced synchrotron structures (*θ*_NO2_ = 69 ± 2° for a synchrotron structure of *Af*NIR) (30). Considering that radiation damages are almost negligible in neutron crystallography, the present NO_2_^-^ ligand data reflect its damage-free state. The vertical binding mode agrees with an earlier report that replacement of ^14^NO_2_^-^ with ^15^NO_2_^-^ on the T2Cu center shows no isotope effect in the electron nuclear double resonance spectra of CuNIR from *Achromobacter xylosoxidans* and NO_2_^-^ hence binds to the T2Cu center only through the O atoms (31).

### Protonation states of the catalytic site in the presence of NO_2_^-^

**Figure 2:**
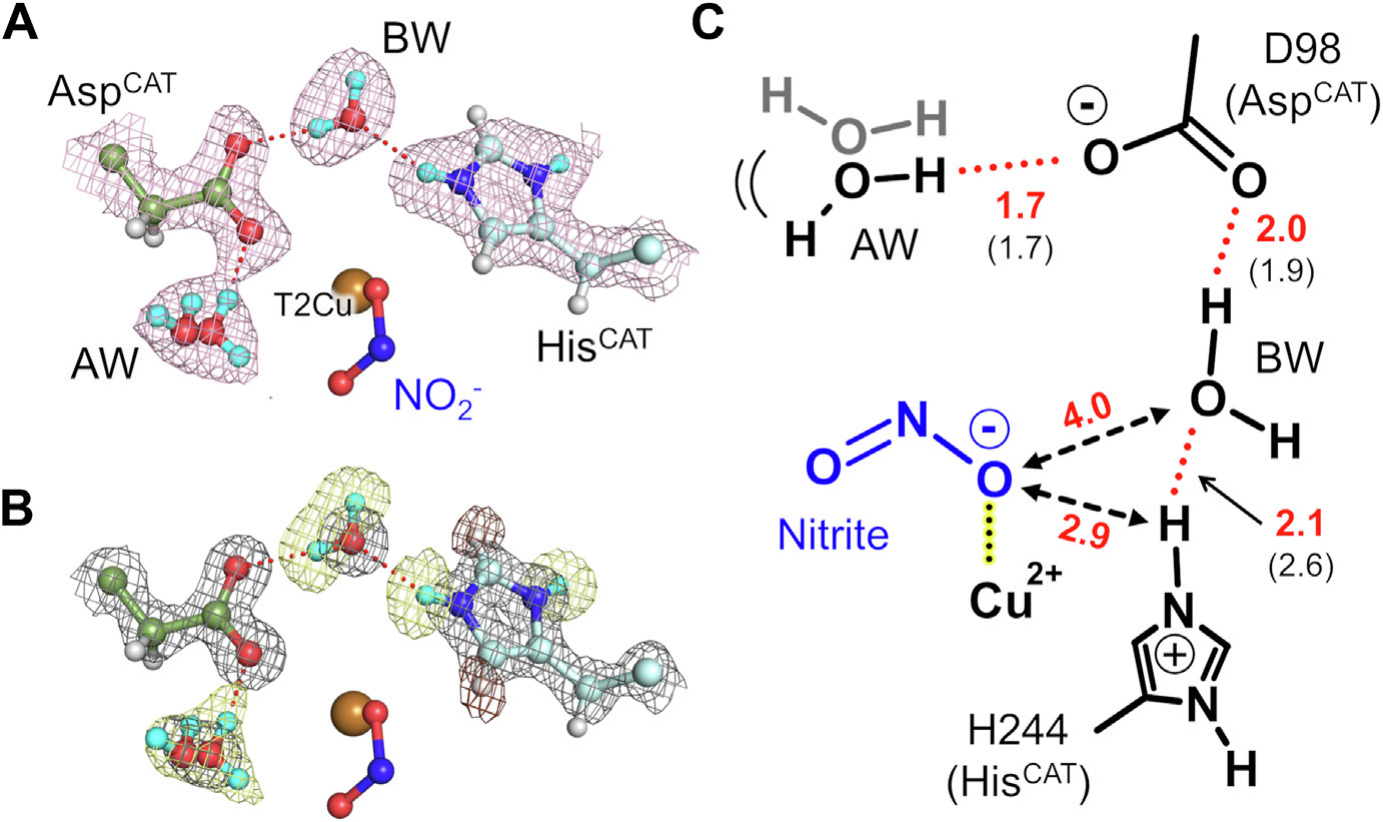
**Hydrogen bond network around the catalytic residues.***A*, sigma-A–weighted 2*F*_o_–*F*_c_ maps for neutron data (*pink*, contoured at 1.0σ). *Dashed red lines* indicate H bonds. *B*, confirmation of the H atom positions. Sigma-A–weighted 2*F*_o_–*F*_c_ maps for X-ray data are shown by *gray meshes* contoured at 2σ. Sigma-A–weighted *F*_o_–*F*_c_ neutron maps are shown by *yellow* (+3.0σ) and *brown* (−3.0σ) *meshes*. H and D atoms are colored by *white* and *cyan*, respectively. A low-occupancy water molecule at the nitrite position has been eliminated for clarity. *C*, the schematics of the hydrogen bond network around the T2Cu site. Distances are shown in Å unit. Distances in parentheses are for the resting state (PDB code: 6L46). T2Cu, type 2 copper.

The 2*mF*_o_-*DF*_c_ neutron-scattering length density map shows D atoms on the two N atoms in the imidazole ring of His^CAT^ (Fig. 2, *A* and *B*), showing that His^CAT^ is protonated in the substrate-bound state at pD5.9. The positive peaks in the *mF*_o_-*DF*_c_ neutron-scattering length density omit map further confirm the presence of D atoms on the imidazole ring of His^CAT^. In contrast, Asp^CAT^ is in the deprotonated state. Asp^CAT^ forms an H-bond with an adjacent water molecule. The electron density and the neutron-scattering length density maps reveal that adjacent water is disordered and adopts at least two conformations. A hydronium (H_3_O^+^) ion could be modeled here but the dual conformation model of water fits the experimental data better. The positions of the D atoms of BW were also determined as illustrated in Figure 2*B*. The H-bond network formed by deprotonated Asp^CAT^, BW, and protonated His^CAT^ is similar to that in the resting state; however, BW is slightly closer to His^CAT^ (Fig. 2*C*) because of subtle rotation of the imidazole ring of His^CAT^ (Fig. S3), which is a known structural change upon NO_2_^-^ binding (30). Rotation of the imidazole ring of His^CAT^ is thought to be a trigger of PT to BW. The shorter H-bond between His^CAT^ and BW may facilitate PT.

The protonation states of the catalytic residues and the presence of OH^-^ at the T2Cu site in our previous resting state neutron structure have been questioned (32) because the structure contains Na^+^ and Cu^2+^ with low occupancies (contaminants from the crystallization solution) above the T2Cu site. In contrast, our present neutron structure lacks extra metal ions at the T2Cu site, visualizing the H-bond network at the catalytic site of *Gt*NIR unquestionably.

### RT neutron structure of the formate-bound state

We previously reported that the differences in temperatures during X-ray data collection affect the NO_2_^-^ binding mode in *Gt*NIR. NO_2_^-^ assumes the monodentate binding mode in cryogenic experiments but the bidentate mode at higher temperatures (33). We therefore attempted RT neutron crystallography to re-evaluate why noncryogenic temperatures alter the substrate binding mode.

We bypassed the problem that NO_2_^-^ decomposes during RT neutron data collection by using formic acid, a stable analog of NO_2_^-^ and an inhibitor of NO_2_^-^ reduction (34). RT ND and XRD data up to 1.90 and 1.20 Å resolution, respectively, were collected from a single crystal of C135A *Gt*NIR in complex with deuterated formic acid (Table S2) and we determined the structure. The electron density shows that formate binds to the T2Cu atom, although it has low occupancy (0.54) (Fig. S4*A*). The corresponding neutron-scattering length density map is somewhat ambiguous (Fig. S4*B*). Calculations using other occupancy values for formate and water molecules showed residual densities (Fig. S4, *C* and *D*). The D atom on the central C atom of formate cannot be visualized in the neutron-scattering length density map although the omit map implies its presence, indicating that the formate ligand is unstable and its position at the T2Cu site fluctuates. Formate assumes a κ^1^-O mode, which is observed in a previously reported cryogenic structure (24) (Fig. S4*E*). This finding suggests that differences of data collection temperature do not affect the binding mode of indecomposable ligands. Therefore, the difference of the NO_2_^-^ binding manners is probably caused by X-ray photoreduction that is greatly facilitated at higher temperatures. The reduction-coupled structural change from the vertical to the near-face-on mode has been observed in several CuNIRs (22, 30). Data collection without cryo-cooling therefore severely reduces the T2Cu center even in the C135A mutant, thereby changing the coordination manners of NO_2_^-^ in *Gt*NIR. In fact, NO_2_^-^ in the *Gt*NIR structure determined at 320 K shows an obvious near-face-on mode with *θ*_NO2_ of 81° (33).

OH^-^ is present on one of the two axial sites of the T2Cu in the resting and NO_2_^-^-bound states, but is partially replaced by a water molecule in the formate-bound structure (Fig. S4, *B* and *F*). This finding means that the ligand at this position can easily absorb and desorb H^+^ at RT and interconvert between OH^-^ and H_2_O. Protons on the interconvertible OH^-^/H_2_O ligand in the present neutron structure are located at 2.2 to 2.6 Å from T2Cu, which shows good agreement with an electron nuclear double resonance study estimating that an exchangeable proton on a T2Cu ligand is located 2.25 to 2.45 Å apart from T2Cu (35).

### Atomic resolution structures of D98N mutants

To further obtain insights into the catalytic intermediates of CuNIR, we performed atomic resolution X-ray crystallography with D98N mutant series of *Gt*NIR in which the Asn residue imitates the protonated state of Asp^CAT^. Pioneering studies on *Af*NIR showed that the replacement of Asp^CAT^ with Asn decreases catalytic activity by two orders of magnitude (17) and changes the binding modes of NO_2_^-^ (36).

The structures of D98N without NO_2_^-^ and C135A/D98N with NO_2_^-^ were determined at 1.05 and 1.12 Å resolution, respectively (Table S3). The NO_2_^-^ complex structure shows multiple conformations of NO_2_^-^ (Fig. S5*A*), indicating that Asp^CAT^ is necessary to keep the substrate at the proper position with the configuration suitable for NO_2_^-^ reduction. A previous 1.65 Å resolution *Af*NIR structure has implied that NO_2_^-^ binds in multiple conformations in the Asn mutant, but the diffuse electron density made modeling of multiple conformers difficult (36). In contrast, our atomic resolution data unambiguously revealed three binding modes of NO_2_^-^. One is a κ^1^-O complex that is present only when Asn98 assumes a conformation similar to the gatekeeper (GK) mode. The GK conformation of Asp^CAT^ was first identified in a CuNIR from *Achromobacter cycloclastes* (37) but has never been found in many other CuNIRs. In the GK-like conformation, the side chain of Asn98 is flipped away from the catalytic site and cannot form a hydrogen bond with the substrate. A GK-like conformation of D98N is not observed in the D98N structure without NO_2_^-^ (Fig. S5*B*), which indicates that repulsion between NO_2_^-^ and Asn98 results in the GK-like conformation.

**Figure 3:**
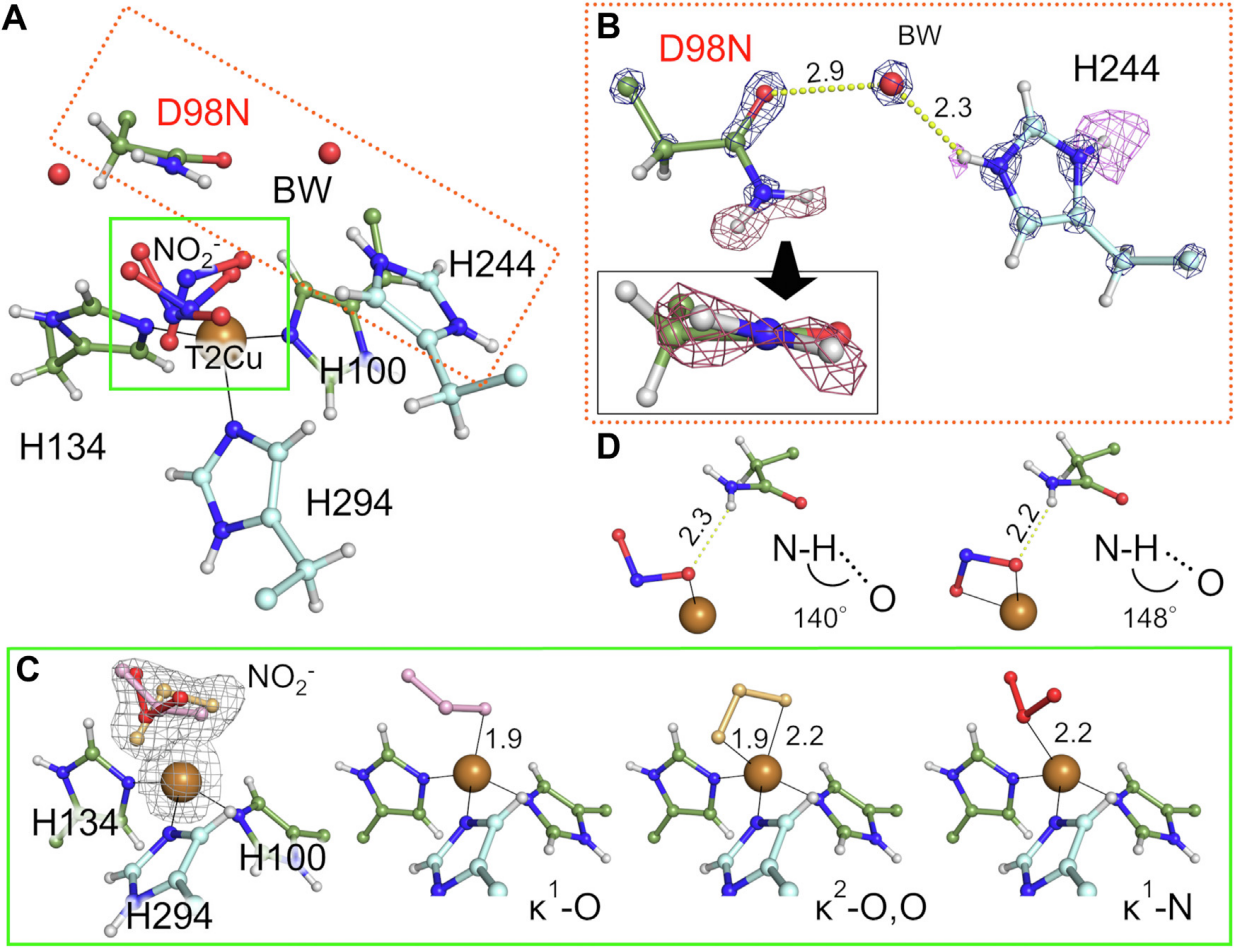
**Structure of the D98N/C135A/G136A mutant.***A*, the T2Cu site of the D98N/C135A/G136A mutant in complex with nitrite. *B*, hydrogen positions of D98N and H244. Sigma-A–weighted 2*F*_o_–*F*_c_ (*gray*, contoured at 5.5σ) and *F*_o_-*F*_c_ (*raspberry*, contoured at 2.5σ; *pink*, contoured at 2.2σ) maps for X-ray data are shown by *meshes*. *C*, three nitrite binding modes observed in the D98N/C135A/G136A mutant. Sigma-A–weighted 2*F*_o_–*F*_c_ maps for X-ray (contoured at 1.0σ) are shown by *gray meshes*. *Black lines* indicate coordination bonds. A low-occupancy water molecule and chloride at the nitrite position have been eliminated for clarity. *D*, interaction between D98N and nitrite. *Dashed yellow lines* indicate H bonds. Distances are shown in Å unit. T2Cu, type 2 copper.

Since the GK-like conformer complicates the interpretation of the T2Cu site structure, we constructed D98N/G136A and D98N/C135A/G136A mutants. We expected that replacing Gly136 with alanine would cause steric hindrance between the side chain methyl group of Ala136 and the GK-like state of Asn98. The structures of D98N/G136A without NO_2_^-^ and D98N/C135A/G136A in complex with NO_2_^-^ were determined at 0.96 and 0.99 Å resolution, respectively (Fig. S5*C* and Table S3). These sub-ångström resolution data depict H atom positions on Asn98 and His^CAT^ (Fig. 3, *A* and *B*), although the H atom positions of BW were ambiguous. The two H atoms of the Asn98 side chain are not on the carbamoyl plane but rather are oriented toward the ligand molecule on the T2Cu atom. This indicates that Asn98 (and perhaps also protonated Asp98) forms an H-bond with the T2Cu axial ligand. As was expected, Asn98 does not exhibit the GK-like conformation in either of the G136A-containing mutants (Fig. 3*A*). However, NO_2_^-^ again shows several coordination manners in the D98N/C135A/G136A structure (Fig. 3*C*), indicating that the GK-like conformation observed in the D98N/C135A mutant is not responsible for the multiple conformers of NO_2_^-^. These observations further support the role of Asp^CAT^ in capturing the substrate on the T2Cu site.

The first conformation of NO_2_^-^ in the D98N/C135A/G136A structure is the κ^1^-O mode that is observed in the native T2Cu site. In this state, the distance between NO_2_^-^ and the amide N atom of Asn98 is 3.2 Å, which is identical to that between Asp^CAT^ and NO_2_^-^ observed in the neutron structure. The second binding mode is the complete κ^2^-O,O coordination in the vertical mode. This state shows a shorter distance to the side chain amide N atom of Asn98 (2.9 Å) than that in the neutron structure (Fig. 3*D*). In the κ^1^-O state, the angle formed by N^N98^-H^N98^-O^nitrite^ is about 140°, which is deviated from the ideal H-bond angle (∼180°). This deviation from ideality is slightly abated in the κ^2^-O,O state (∼148°). Because the complete κ^2^-O,O bidentate mode is typical of the D98N mutants, the hydrogen bond with Asp^CAT^ is necessary for stabilization of the κ^2^-O,O mode. The third binding mode is the κ^1^-N mode that is also observed in the C135A/D98N structure. The κ^1^-N mode appears in the model complexes of CuNIR (13), although no protein structures supporting the presence of the κ^1^-N mode during the CuNIR catalytic cycle are currently available. Since the κ^1^-N mode has not been observed in the WT enzyme, the catalytic site of CuNIR may be designed to avoid the κ^1^-N mode.

### Computational chemistry based on the neutron structure

**Figure 4:**
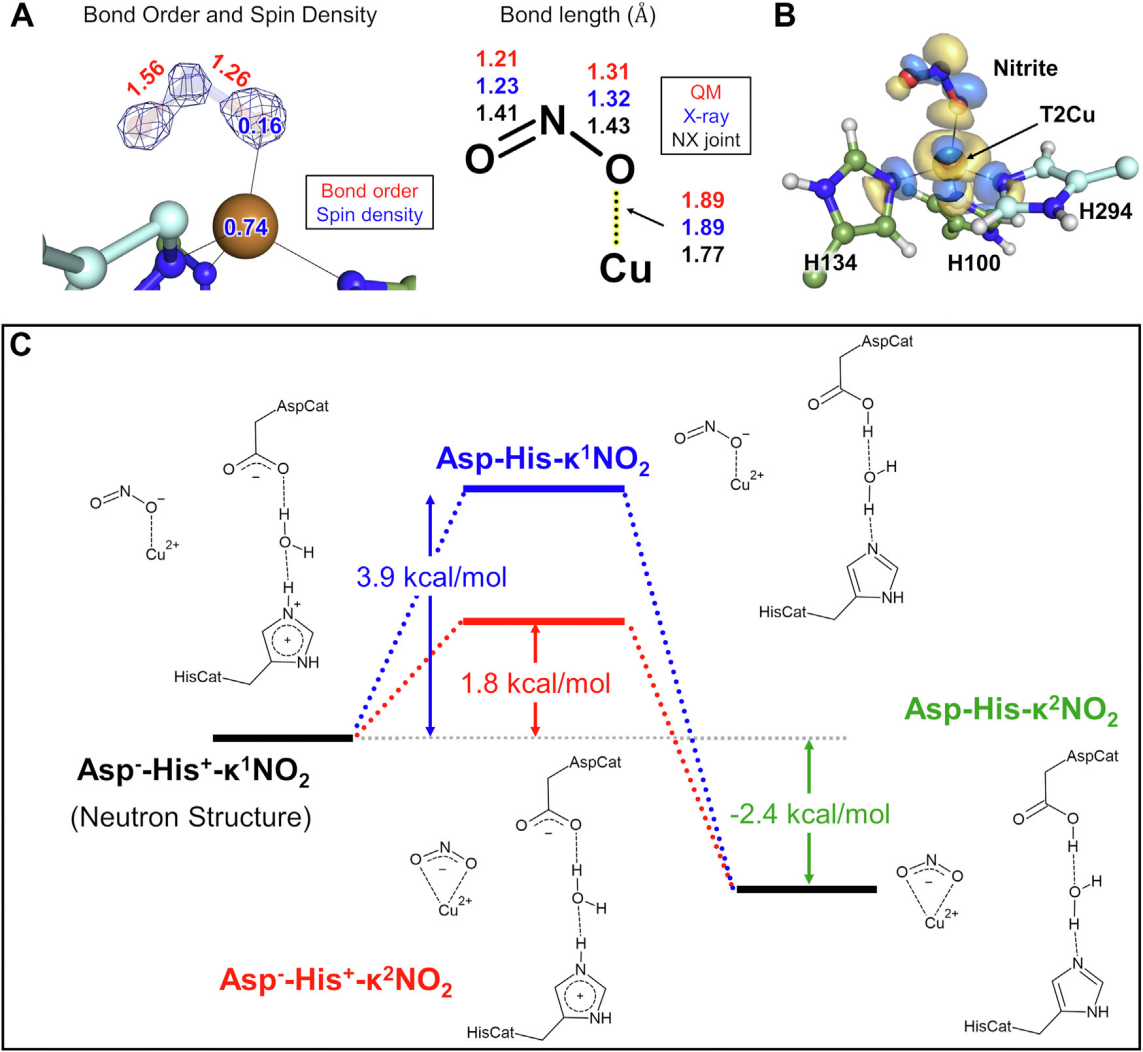
**Computational analysis.***A*, asymmetric structure of nitrite. The nitrite-omit *mF*_o_-*DF*_c_ electron density map (+13.3σ) is overlayed on the model structure. An alternative water molecule located at the same position as nitrite was included in the model when the omit map was calculated. Spin density values were obtained by Mulliken population analysis. Bond lengths for the X-ray structure are from refinement against the X-ray data. *B*, the lowest unoccupied molecular orbital (LUMO) of the T2Cu site calculated based on the neutron structure. Coordination bonds are represented by *black lines*. *C*, Gibbs free energy changes during structural changes from the Asp^-^-His^+^-κ^1^NO_2_^-^ state at 100 K. T2Cu, type 2 copper.

To evaluate our neutron structure in complex with NO_2_^-^, we performed quantum mechanics/molecular mechanics (QM/MM) calculations. Analyses with the neutron structure and a computationally optimized structure both show localization of the spin density of the catalytic center on the T2Cu atom, supporting that our experimental structure is in the Cu(II) state (Fig. 4*A*) and hence that we obtained a damage-free structure with neutron crystallography. The calculation suggested that the bond lengths and the bond orders of the two N-O bonds in NO_2_^-^ differed (Fig. 4, *A* and *B*), and that the NO_2_^-^ ligand on the oxidized T2Cu center can be described as O=N-O^-^. We verified this result by refining the NO_2_^-^-bound structure using the X-ray data up to 1.0 Å resolution: the electron density shows the difference between the single and double bonds in NO_2_^-^, supporting the localization of electrons (Fig. 4*A*).

The lowest unoccupied molecular orbital (LUMO) of the κ^1^ NO_2_^-^-bound state is an antibonding orbital mainly composed of the d_*z*^2^_ orbital of the T2Cu atom, lone pairs of His ligands, and a lone pair of one of the O atoms of NO_2_^-^ (Fig. 4*C*). The antibonding orbital of one of the N-O bonds in NO_2_^-^, which is distant from the T2Cu atom, also slightly contributes to the LUMO. This molecular orbital is similar to the LUMO previously calculated for the κ^2^ NO_2_^-^-bound state of CuNIR from *Rhodobacter sphaeroides* (38).

QM/MM analysis using a small QM region (T2Cu, His ligands, and several water molecules including BW) shows that the Asp-His-κ^1^NO_2_^-^ state, in which Asp^CAT^ is protonated and His^CAT^ is deprotonated, is much stable (Δ*G* = −18.9 kcal/mol) than the Asp^-^-His^+^-κ^1^NO_2_^-^ state observed in the neutron structure (Fig. S6). The Asp^-^-His^+^-κ^1^NO_2_^-^ state is thus converted to the Asp-His-κ^1^NO_2_^-^ state without having to cross an energy barrier. However, this result is inconsistent with the experimental data, indicating that other factors stabilize the Asp^-^-His^+^-κ^1^NO_2_^-^ state.

One such factor is the residue pair Gln267 and Thr268. The Gln267/Thr268 pair can form a hydrogen bond with His^CAT^ (Figs. 1*A* and S7) and is proposed to function as a redox-coupled proton switch (30). Energy differences calculated by using the QM region including the Gln267/Thr268 pair show an equilibrium between the Asp^-^-His^+^-κ^1^NO_2_^-^ and the Asp-His-κ^1^NO_2_^-^ states with an energy barrier of a few kcal per mol between them. This result indicates that the H-bond between His^CAT^ and Gln267 is important for maintaining a proton on His^CAT^. However, the Asp^-^-His^+^-κ^1^NO_2_^-^ state observed in the neutron structure is still less stable than the Asp-His-κ^1^NO_2_^-^ state (Δ*G* = −8.4 kcal/mol; Fig. S8).

A second factor is the relative permittivity of solvent molecules. Because the T2Cu site is located at an interface of two protomers and the water/proton channel from the molecular surface to the T2Cu site is filled with solvent molecules, we hypothesized that the relative permittivity of solvent molecules around the catalytic site affects the stability of the highly polarized Asp^-^-His^+^-κ^1^NO_2_^-^ state. Inclusion of the solvent effect by using the polarizable continuum model shows that the Asp^-^-His^+^-κ^1^NO_2_^-^ state is more stable than the Asp-His-κ^1^NO_2_^-^ state at 100 K, the temperature at which we collected the crystallographic data (Fig. 4*C*). This result shows that the substrate-binding state observed in the neutron structure is stabilized by the solvated environment.

After including the effects of the Gln267/Thr268 pair and the relative permittivity of solvent molecules, we calculated the free energy changes at a more physiological temperature (300 K) and observed that the Asp^-^-His^+^-κ^1^NO_2_^-^ state coexists with the Asp-His-κ^1^NO_2_^-^, Asp^-^-His^+^-κ^2^NO_2_, and Asp-His-κ^2^NO_2_ states (Fig. S9). The oxidized T2Cu site in complex with NO_2_^-^ thus moves back and forth between these three states in physiological environments.

## Discussion

NO_2_^-^ reduction in CuNIR can follow two routes (39). In one route, NO_2_^-^ binds to the oxidized T2Cu site prior to intramolecular ET from T1Cu to T2Cu. In the other route, T2Cu is reduced before it captures NO_2_^-^. We here focus on the first route because we obtained the neutron structure exhibiting NO_2_^-^ on the oxidized T2Cu site. Several reports consider this route as being the main route because the prereduced T2Cu site reacts with the substrate very slowly (40) and intramolecular ET before NO_2_^-^ binding is energetically unfavorable (41). Moreover, this route shows an intriguing “gating by NO_2_^-^” feature; that is, the binding of NO_2_^-^ on the oxidized T2Cu site accelerates intramolecular ET from T1Cu to T2Cu (19, 21, 42).

Neutron crystallography revealed that NO_2_^-^ at the initial binding stage is in a deprotonated and electron-localized state. Although previously proposed mechanisms of the CuNIR reaction have often described NO_2_^-^ as HNO_2_ when it binds to the oxidized T2Cu site, our direct observation proved that substrate binding itself does not induce PT to the substrate. Neutron crystallography also showed that Asp^CAT^ is deprotonated and His^CAT^ is protonated in the substrate-bound oxidized state. This Asp^-^-His^+^-κ^1^NO_2_^-^ state of the enzyme–substrate complex is stabilized by the relative permittivity derived from solvent molecules filling the catalytic site and the H-bond between His^CAT^ and Gln267. The T2Cu center is constructed at the protomer interface through the evolution of copper-containing enzymes (43). The importance of permittivity in formation of an unstable enzyme–substrate complex can explain why copper-containing enzymes have evolved with their catalytic center at the protomer interface where solvent molecules easily access.

The combination of neutron crystallography and QM/MM calculations showed that the catalytic site is generally in the Asp^-^-His^+^ state. Protonated His^CAT^ indicates that His^CAT^ initiates the PT sequence. PT triggers ET from T1Cu to T2Cu (44) and the proton-coupled ET reaction is facilitated by NO_2_^-^ binding (19, 21, 42). QM/MM calculations revealed that the oxidized T2Cu site in complex with NO_2_^-^ adopts a mixture of different protonation states. The equilibrium condition shifts toward a state in which His^CAT^ is deprotonated upon reduction of the T2Cu site. NO_2_^-^ subsequently receives an H^+^ from BW or from Asp^CAT^ *via* a proton relay around the catalytic site. Simultaneously, NO_2_^-^ undergoes a structural change to the near-face-on manner on the reduced T2Cu site (30), where it forms an H-bond with Asp^CAT^ to stabilize the transient intermediate, as observed in the D98N mutant structures. This protonation process of NO_2_^-^ helps ET from T2Cu to NO_2_^-^ because protonation can lower the energy level of the antibonding orbital of the substrate, allowing interaction with the Cu *d* orbital (38). The electron-localized state of NO_2_^-^ can facilitate the generation of NO. An electron entering the antibonding orbital weakens the single-bond-like N-O bond in NO_2_^-^, leading ultimately to the formation of NO and OH^-^. The NO molecule is released and OH^-^ remains on the T2Cu site. In this OH-bound state, ET from T1Cu to T2Cu is much slower than in the water-bound and NO_2_^-^-bound states (39), inhibiting undesired reactions such as oxygen reduction, which causes selfinactivation of CuNIR (45), before the next NO_2_^-^ ion approaches T2Cu. The resting state is reconstructed when the T2Cu site receives another proton from the water/proton channel.

## Conclusion

We determined the high-resolution neutron structure of *Gt*NIR in complex with NO_2_^-^ as well as the subatomic resolution X-ray structures of D98N mutant series with and without bound NO_2_^-^. Based on the experimental structure including H atoms, we performed QM/MM calculations to investigate the dynamics of H atoms. Obtained results help us to understand the CuNIR reaction in detail, showing the potential of neutron crystallography to scrutinize chemical reactions coupled with PT on metal centers at atomic and even quantum levels.

Less than 0.5% of all crystal structures deposited in the structural databases (the Cambridge Structural Database, the Inorganic Crystal Structure Database, and the Protein Data Bank [PDB]) have been solved by the ND method (46), hampering our understanding of the chemical behavior of H atoms in organic and inorganic materials. Chemical reactions involving nitrogen oxides on copper in small compounds (47, 48, 49) and *de novo* artificial enzymes (50) are recent trends in the chemistry field. Our study provided the first biologically relevant nitrito–copper complex structure determined by ND crystallography; therefore, it can contribute to designing new catalysts for nitrogen oxide chemistry. Determining other single-hydrogen-level structures of CuNIR such as an NO complex and a Cu(I) state will lead to further progress of understanding not only CuNIR but also related chemical reactions.

## Experimental procedures

*Gt*NIR crystals were prepared as described previously (14). For ND data collection, the obtained crystals were placed in the deuterated solution and soaked in a freshly prepared solution containing 200 mM NaNO_2_ or 50 mM deuterated ammonium formate. The crystal for cryogenic neutron crystallography was flash-cooled in a nitrogen-gas stream. ND data collection was performed at a beamline BL03 iBIX in the Materials and Life Sciences Experimental Facility of the Japan Proton Accelerator Research Complex (51). X-ray data for NX joint refinement were collected at a beamline AR-NW12 of Photon Factory Advanced Ring at 100 K and a beamline BL5A of Photon Factory at RT. X-ray data of *Gt*NIR D98N mutants were collected at a beamline BL44XU of SPring-8. Data processing and structural analysis were performed as described in SI Appendix, Materials and Methods. The QM/MM calculations were performed by using the two layer ONIOM scheme (52). For QM region, B3LYP was used with the 6-31G(d,p), and the Amber force field (53) was used for the MM region. All calculations were carried out by using Gaussian 16 (54). Complete methods are described in Experimental procedures of Supporting information.

## Data availability

The raw diffraction data collected in this study are available at the Xtal Raw Data Archive (https://xrda.pdbj.org) under the IDs corresponding to the PDB depositions. The coordinate files and the structure factor files are deposited in the PDB (PDB IDs: 9KVL for the NO_2_^-^ complex neutron structure, 9KVM for the formate complex neutron structure, 9KWS for the X-ray structure of the D98N mutant, 9KWU for the X-ray structure of the D98N/C135A mutant in complex with NO_2_^-^, 9KWT for the X-ray structure of the D98N/G136A mutant in complex with NO_2_^-^, and 9KWV for the X-ray structure of the D98N/C135A/G136A mutant in complex with NO_2_^-^).

## Supporting information

This article contains supporting information (14, 51, 52, 53, 54, 55, 56, 57, 58, 59, 60, 61, 62).

## Conflicts of interest

The authors declare that they have no conflicts of interest with the contents of this article.
