# Supplementary material for: Structural basis of cuproenzyme nitrite reduction at the level of a single hydrogen atom
Source: J Biol Chem. 2025 May 26;301(7):110290. doi: 10.1016/j.jbc.2025.110290 (PMC12221283; doi:10.1016/j.jbc.2025.110290)
Supplement: Supporting Information [file mmc1.docx]

**Supporting Information for**

**Structural basis of cuproenzyme nitrite reduction**

**at the level of a single hydrogen atom**

**Authors:** Yohta Fukuda, Masami Lintuluoto, Yu Hirano, Katsuhiro Kusaka, Tsuyoshi Inoue, Taro Tamada

**1. Complete Materials and Methods (pp. 2-6)**

1.1. Expression and purification of *Gt*NIR mutant proteins.

1.2. Preparing large crystals of the C135A mutant.

1.3. Cryogenic neutron and X-ray data collection of C135A-NaNO_2_.

1.4. Room temperature neutron and X-ray data collection of C135A-formate.

1.5. Structural determination and NX-joint refinement.

1.6. Atomic resolution X-ray diffraction data collection and data processing for C135A/D98N mutant series.

1.7. Structural determination and refinement for C135A/D98N mutant series.

1.8. Computational methods.

**3. SI Tables (pp. 7-9)**

Table S1. Data collection and refinement statistics for C135A-NO_2_.

Table S2. Data collection and refinement statistics for C135A-formate.

Table S3. Data collection and refinement statistics for C135A series.

**4. SI Figures (pp. 10-18)**

Fig. S1. Ligands at the T2Cu site in the neutron structure of the C135A-nitrite complex.

Fig. S2. Comparison between the cryogenic neutron structure and the X-ray structure determined at 320 K.

Fig. S3. Rotation of the imidazole ring of His^CAT^.

Fig. S4. Neutron structure of C135A *Gt*NIR in complex with formate.

Fig. S5. Structures of D98N *Gt*NIR mutants.

Fig. S6. Energy differences calculated by using a small QM region.

Fig. S7. Close-up view of a redox-coupled proton switch (Q267 and T268).

Fig. S8. Energy differences calculated by using a model including the Gln/Thr pair but without a solvent effect.

Fig. S9. Energy differences calculated by considering the Gln/Thr pair and a solvent effect at 300 K.

**5. SI References (pp. 19)**

1. **Complete Materials and Methods**
   1. **Expression and purification of *Gt*NIR mutant proteins.**

All mutant proteins are prepared by the same protocol as described below. *Gt*NIR mutant proteins were expressed and purified according to the method described previously (14). A plasmid (pET22b) containing genes of *Gt*NIR mutants were transformed into E. coli BL21(DE3) strain. Expression of the proteins was induced by 1 mM IPTG at 20 °C for 18 h. The cells were sonicated in buffer A (20 mM Tris-HCl, pH 8.0) and incubated at 70 °C for 120 min. The sample was centrifuged, 10 mM CuSO_4_ was added to the supernatant until the colour of the solution turned deep green. The resulting solution was loaded onto a HiLoad 16/600 Superdex 200 column (GE Healthcare). The fractions containing *Gt*NIR were collected and ammonium sulfate was added up to 40% w/v. After incubation at 4 °C for 30 min, the solution was centrifuged and the supernatant was applied onto a HiTrap Phenyl HP column (GE Healthcare). The protein was eluted with a linear gradient of ammonium sulfate, dialyzed against buffer A, and purified with a HiTrap Q HP column using sodium chloride. The fractions containing *Gt*NIR were then loaded onto a HiLoad 16/60 Superdex 200 column equilibrated by buffer A. The peak fractions were collected and concentrated to 90 mg/ml.

**1.2. Preparing large crystals of the C135A mutant.**

Large *Gt*NIR crystals were prepared by a combination of micro- and macro-seeding methods. First, crystals were obtained at 20 °C by the sitting-drop vapor-diffusion method with a 1:1 mixture of the purified protein solution (30 ~ 45 mg/ml) and a reservoir solution containing 0.1 M acetate buffer pH 4.5, 5.0 % (w/v) PEG 4000, and 75 mM CuSO_4_. Obtained crystals were shattered in a drop by a needle. The resulting solution was 10 times diluted by the reservoir solution to make a seed solution. Streak microseeding crystallization was performed at 20 °C by the hanging-drop vapor-diffusion method. We dipped a single hair obtained from one of the authors (a male individual of Homo sapiens) into the seed solution and then run it through the drop of the mixture of purified protein (30 mg/ml; 1.5 μL) and a reservoir solution (0.1 M acetate buffer pH 4.5, 5.5 % (w/v) PEG 4000, and 75 mM CuSO4; 1.5 μL) on a siliconized cover glass. The cover glass was put on a 0.5 mL sample cups (Sanplatec) containing 400 μL reservoir solution. At this microseeding crystallization step, high-quality small single crystals could be obtained within a day.

Macroseeding crystallization was performed at 20 °C by the floating-drop vapor-diffusion method. The purified protein solution (30 ~ 50 mg/ml; 50 ~ 200 μL) was mixed with a reservoir solution (0.1 M acetate buffer pH 4.6 ~ 4.8, 5.5 % (w/v) PEG 4000, and 50 mM CuSO_4_; 50 ~ 200 μL) and transferred onto a micro-bridge (Hampton research) or to an inner dent of a snap cap of a Falcon 14 mL polypropylene round-bottom tube (Corning), which were half-filled with fluorinert (Hampton research) to prevent grown crystals from sticking to the bottom. In the former case, the micro-brides were set in a 24-well VDX plate (Hampton research) that contained the reservoir solution of 500 μL. Each well was sealed by a 22 mm × 0.22 mm siliconized circle cover slide (Hampton research) and the plate was left at 20 °C for 2 days to progress vapor diffusion. In the latter case, the outer moat of the snap cap was filled with the reservoir solution. The snap cap was sealed by a 22 mm × 0.22 mm siliconized circle cover slide and left at 20 °C for 2 days to progress vapor diffusion. Small parallelogram crystals obtained from a microseeding drop were washed several times by the reservoir solution without CuSO_4_ and transferred to the crystallization set-up in the micro-bridges or snap caps with a LithoLoop (Protein Wave).

- 1. **Cryogenic neutron and X-ray data collection of C135A-NaNO_2_.**

After we obtained large-volume crystals, we gradually increased the concentration of D_2_O and PEG in the reservoir by changing the reservoir solutions. At this step, PEG 4000 is replaced with PEG 3350, and CuSO_4_ was not included in the used reservoir solution. At the same time, we gradually increased the concentration of cryo-protectant (2-methyl-2,4-pentanediol: MPD; ethylene glycol). pH was also gradually changed to pH5.5 (pD5.9).

The crystal for data collection was kept in the deuterated solution supplemented with 5 mM NaNO_2_ for 2 week. It was then soaked in the deuterated solution supplemented with 10 mM NaNO_2_ for 1 day. Finally, it was transferred into the deuterated solution supplemented with 100 mM NaNO_2_ 1 day before the data collection. Therefore, the final solution for the cryogenic experiment contained D_2_O, 0.1 M monodeuterated acetate buffer pD 5.9, 8 % (w/v) PEG 3350, 20 % (v/v) perdeuterated MPD, 8 % (v/v) perdeuterated ethylene glycol, and 100 mM NaNO_2_.

The large-volume crystal was harvested by a LithoLoop attached on a B3 magnetic goniometer base (MiTeGen) and flash-cooled in a nitrogen-gas stream at 100 K on a goniometer head at a beamline BL03 iBIX in the Materials and Life Sciences Experimental Facility (MLF) of the Japan Proton Accelerator Research Complex (J-PARC; Tokai, Japan) (51). Time-of-flight (TOF) neutron diffraction data were collected by using thirty wavelength-shifting fiber-based scintillator neutron detectors with an area of 133 × 133 mm^2^. A total of 41 data sets were collected using a wavelength of 2.15-4.89 Å with a sample-to-detector distance of 490 mm. The exposure time for each data set was 6.5 h at 500 kW. This large crystal was also used for cryogenic X-ray diffraction data collection at a beamline AR-NW12 of Photon Factory Advanced Ring (PF-AR; Tsukuba, Japan). Diffraction images were collected at 100 K using a PILATUS3 S2M detector (DECTRIS) with a sample-to-detector distance of 96.651 mm. The wavelength of X-rays was 0.8 Å. The oscillation angle per image was set to 0.1 °. The exposure time per image was set to 0.1 sec. A total of 1800 diffraction images were collected by using a helical scan method to reduce radiation damages.

- 1. **Room temperature neutron and X-ray data collection of C135A-formate.**

After we obtained large-volume crystals, we gradually increased the concentration of D2O and PEG in the reservoir by changing the reservoir solutions. At this step, PEG 4000 is replaced with PEG 3350, and CuSO_4_ was not included in the used reservoir solution. pH was also gradually changed to pH5.6 (pD6.0). The crystal for data collection was kept in the deuterated solution supplemented with 10 mM perdeuterated formate ammonium for 3 days. It was then transferred into the deuterated solution supplemented with 50 mM perdeuterated formate ammonium 1 day before the data collection. Therefore, the final solution for the room temperature experiment contained D_2_O, 0.1 M monodeuterated acetate buffer pD 6.0, 6.5 % (w/v) PEG 3350, and 50 mM formate. The crystal was harvested by a LithoLoop and transferred into a quartz capillary (Hampton Research). The crystal was surrounded by a small amount of crystallization solution and the capillary was sealed with wax (Hampton Research) to prevent desiccation of the crystal.

The capillary containing the crystal was set on a goniometer head at a beamline BL03 iBIX in the Materials and Life Sciences Experimental Facility (MLF) of the Japan Proton Accelerator Research Complex (J-PARC; Tokai, Ibaraki, Japan) at room temperature. Time-of-flight (TOF) neutron diffraction data were collected by using thirty wavelength-shifting fiber-based scintillator neutron detectors with an area of 133 × 133 mm^2^. A total of 44 data sets were collected using a wavelength of 2.44-5.28 Å with a sample-to-detector distance of 490 mm. The exposure time for each data set was 5.5 h at 740 kW. This large crystal was also used for cryogenic X-ray diffraction data collection at a beamline BL5A of Photon Factory (Tsukuba, Ibaraki, Japan). Diffraction images were collected at room temperature using a Pilatus3 S6M detector (DECTRIS) with a sample-to-detector distance of 122.28 mm. The wavelength of X-rays was 1.0 Å. The oscillation angle per image was set to 0.1 °. The exposure time per image was set to 0.1 sec. A total of 1800 diffraction images were collected by using a helical scan method to reduce radiation damages.

- 1. **Structural determination and NX-joint refinement.**

The TOF neutron data were integrated with STARGazer (55) with profile fitting (elliptic) (56). The data were then merged and scaled by SCALA (57). The X-ray diffraction data were processed and scaled by using XDS (58) and AIMLESS, respectively. Molecular replacement phase determination was performed by MOLREP (59) with a *Gt*NIR structure (PDB code ID: 4YSO) as a search model. The joint refinement was performed with both the neutron and X-ray diffraction data using phenix.refine implemented in PHENIX (60). Five percent of the data were selected by PHENIX for cross-validation. Manual model building was performed using COOT (61) through which positions of H (D) atoms and H^+^ (D^+^) on amino acid residues and orientation of water molecules were manually modeled consulting both the neutron-scattering length density and electron density calculated before including hydrogen/deuterium. The temperature factors for all atoms and occupancies for hydrogen/deuterium atoms and residues having dual conformations were also refined. The final model quality was checked by MolProbity (62).

- 1. **Atomic resolution X-ray diffraction data collection and data processing for C135A/D98N mutant series.**

The crystals were harvested by LithoLoops, flash-cooled in liquid nitrogen, and set on the gonio head equipped at BL44XU of SPring-8 (Sayo, Hyogo, Japan). Diffraction images were collected at 100 K using an EIGER 16M detector (DECTRIS). The wavelength of X-ray was 0.9 Å. The oscillation angle per image was set to 0.1 °. The exposure time per image was set to 1 sec. The transmittance of X-ray was set to 0.1. A total of 1800 diffraction images were collected for each sample. The obtained X-ray diffraction data were processed and scaled by using XDS and AIMLESS, respectively.

- 1. **Structural determination and refinement for C135A/D98N mutant series.**

Molecular replacement phase determination was performed by MOLREP with a GtNIR structure (PDB code ID: 4YSO) as a search model. The refinement was performed with phenix.refine implemented in PHENIX. Five percent of the data were selected by PHENIX for *R*_free_ cross-validation. Manual model building was performed using COOT through which positions of H atoms on amino acid residues and orientation of water molecules were manually modeled. The temperature factors for all atoms and occupancies for H atoms and residues having dual conformations were also refined. The final model quality was checked by MolProbity.

- 1. **Computational methods.**

Initial coordinates of GtNIR were derived from the neutron diffraction data. The QM/MM calculations were performed by using the two layer ONIOM scheme (52). For QM region, B3LYP was used with the 6-31G(d,p), and the Amber force field (53) was used for the MM region. The QM region included T2 Cu atom, His ligands, two catalytic amino acid residues, and three water molecules were also included in QM region. Other amino residues were included in MM region. The structural optimizations were carried out by using QM/MM models. We estimated the Gibbs free energy changes along with the structural change by using the small QM models including only QM region of QM/MM models. All calculations were carried out by using Gaussian 16 (54).

**SI Tables**

| Table S1 \| Data collection and refinement statistics for C135A-NO_2_ | | |
| --- | --- | --- |
|  | **Neutron** | **X-ray** |
| Data collection | | |
| Beamline | J-PARC MLF BL03 iBIX | Photon Factory AR-NW12 |
| Wavelength (Å) | 4.89-2.15 | 0.8 |
| Space group | *R*3 (*H*3) | |
| Unit cell *a*, *b, c* (Å) | 115.06, 115.06, 84.48 (*H*3) | |
| Resolution range (Å) | 20.00-1.70 (1.79-1.70) | 42.92-1.00 (1.02-1.00) |
| *R*_merge_ (%) | 24.7 (56.5) | 7.6 (33.0) |
| *R*_p.i.m._ (%) | 9.6 (24.3) | 3.7 (16.2) |
| Completeness (%) | 94.6 (87.1) | 100 (100) |
| Total reflections | 316,988 (34,003) | 1,145,998 (57,451) |
| Unique reflections | 43,445 (5,851) | 225,316 (11,171) |
| <*I*/σ (*I*)> | 8.8 (3.0) | 13.6 (4.6) |
| *CC*_1/2_ | 0.973 (0.778) | 0.996 (0.940) |
| Redundancy | 7.3 (5.8) | 5.1 (5.1) |
| NX Joint Refinement | | |
| Resolution (Å) | 13.20-1.70 (1.74-1.70) | 28.16-1.30 (1.32-1.30) |
| *R*_work_ (%)/ *R*_free_ (%) | 15.6/17.2 (19.4/22.7) | 11.4/13.2 (10.6/14.3) |
| No. of protein atoms | 5,108 | |
| No. of ligand atoms/ions | 78 | |
| No. of D_2_O | 336 (including O and OD types) | |
| Average *B* (Å^2^) |  | |
| All | 16.5 | |
| Protein atoms | 13.5 | |
| D_2_O | 36.4 | |
| Other atoms | 54.5 | |
| Ramachandran plot (%) |  | |
| Favored | 97.3 | |
| Allowed | 2.7 | |
| Outliers | 0 | |
| PDB code ID | 9KVL | |

| Table S2 \| Data collection and refinement statistics for C135A-formate | | |
| --- | --- | --- |
|  | **Neutron** | **X-ray** |
| Data collection | | |
| Beamline | J-PARC MLF BL03 iBIX | Photon Factory BL5A |
| Wavelength (Å) | 5.28-2.90 | 1.0 |
| Space group | *R*3 (*H*3) | |
| Unit cell *a*, *b, c* (Å) | 116.2, 116.2, 85.6 (*H*3) | |
| Resolution range (Å) | 20.0-1.90 (1.59-1.90) | 43.4-1.20 (1.22-1.20) |
| *R*_merge_ (%) | 26.4 (89.0) | 8.8 (90.0) |
| *R*_p.i.m._ (%) | 10.8 (42.8) | 2.1 (25.3) |
| Completeness (%) | 97.7 (99.5) | 4.2 (44.9) |
| Total reflections | 226,653 (25,201) | 680,928 (33,514) |
| Unique reflections | 33,212 (4,926) | 134,752 (6,689) |
| <*I*/σ (*I*)> | 6.5 (1.7) | 9.7 (1.7) |
| *CC*_1/2_ | 0.975 (0.527) | (0.701) |
| Redundancy | 6.8 (5.1) | 5.1 (5.0) |
| NX Joint Refinement | | |
| Resolution (Å) | 12.9-1.90 (1.94-1.90) | 39.4-1.20 (1.21-1.20) |
| *R*_work_ (%)/ *R*_free_ (%) | 14.6/18.3 (23.7/25.3) | 11.4/11.9 (25.0/26.0) |
| No. of protein atoms | 5033 | |
| No. of ligand atoms/ions | 8 | |
| No. of D_2_O | 227 (including O and OD types) | |
| Average *B* (Å^2^) |  | |
| All | 30.0 | |
| Protein atoms | 28.3 | |
| D_2_O | 47.8 | |
| Other atoms | 30.8 | |
| Ramachandran plot (%) |  | |
| Favored | 97.0 | |
| Allowed | 3.0 | |
| Outliers | 0 | |
| PDB code ID | 9KVM | |

| Table S3 \| Data collection and refinement statistics for C135A series | | | | |
| --- | --- | --- | --- | --- |
|  | **D98N** | **D98N/C135A**  **with NO_2_** | **D98N/G136A** | **D98N/C135A/G136A with NO_2_** |
| Data collection at SPring-8 BL44XU using 0.9 Å wavelength X-rays | | | | |
| Space group | *R*3 (*H*3) | | | |
| Unit cell  *a*, *b, c* (Å) | 115.0, 115.0, 84.4 | 115.3, 115.3, 84.3 | 115.1, 115.1, 84.4 | 115.4 115.4 84.3 |
| Resolution range (Å) | 42.9-1.05  (1.07-1.05) | 43.0-1.12  (1.14-1.12) | 43.0-0.96  (0.98-0.96) | 43.0-0.99  (1.01-0.99) |
| *R*_merge_ (%) | 0.049 (0.431) | 0.062 (0.608) | 0.070 (1.127) | 0.067 (1.556) |
| *R*_p.i.m._ (%) | 0.030 (0.277) | 0.037 (0.390) | 0.045 (0.691) | 0.033 (0.749) |
| Completeness (%) | 99.4 (100) | 96.6 (98.9) | 99.7 (100) | 99.8 (100) |
| Total reflections | 647,172  (31,913) | 532,207  (26,684) | 1,300,695  (64,599) | 1,192,852  (61,188) |
| Unique reflections | 193,408 (9,589) | 155,451 (7,879) | 254,215 (12,736) | 232,705 (11,509) |
| <*I*/σ (*I*)> | 11.1 (2.1) | 9.4 (2.0) | 10.0 (1.3) | 10.5 (1.0) |
| *CC*_1/2_ | 0.998 (0.803) | 0.997 (0.617) | 0.999 (0.579) | 0.999 (0.542) |
| Redundancy | 3.3 (3.3) | 3.4 (3.4) | 5.1 (5.1) | 5.1 (5.3) |
| Refinement | | | | |
| Resolution (Å) | 26.26-1.05  (1.09-1.05) | 32.22-1.12  (1.13-1.12) | 28.1-0.96  (0.97-0.96) | 28.84-0.99  (1.00-0.99) |
| *R*_work_ (%)/ *R*_free_ (%) | 8.96/ 10.1 (21.8/22.6) | 10.3/11.7 (29.6/31.1) | 10.0/10.7 (24.6/25.3) | 11.0/12.1 (29.1/28.4) |
| No. of protein atoms | 5,025 | 4651 | 5,735 | 5,293 |
| No. of ligand atoms | 20 | 23 | 30 | 34 |
| No. of H_2_O | 427 | 395 | 421 | 359 |
| Average *B* (Å^2^) |  |  |  |  |
| All | 15.6 | 15.3 | 13.7 | 13.7 |
| Protein atoms | 12.8 | 13.1 | 11.6 | 11.9 |
| H_2_O | 31.2 | 28.9 | 27.2 | 25.1 |
| Other atoms | 26.0 | 18.8 | 23.8 | 20.2 |
| Ramachandran (%) |  |  |  |  |
| Favored | 97.3 | 98.0 | 98.0 | 96.6 |
| Allowed | 2.7 | 2.0 | 2.0 | 3.4 |
| Outliers | 0 | 0.34 | 0 | 0 |
| PDB code ID | 9KWS | 9KWU | 9KWT | 9KWV |


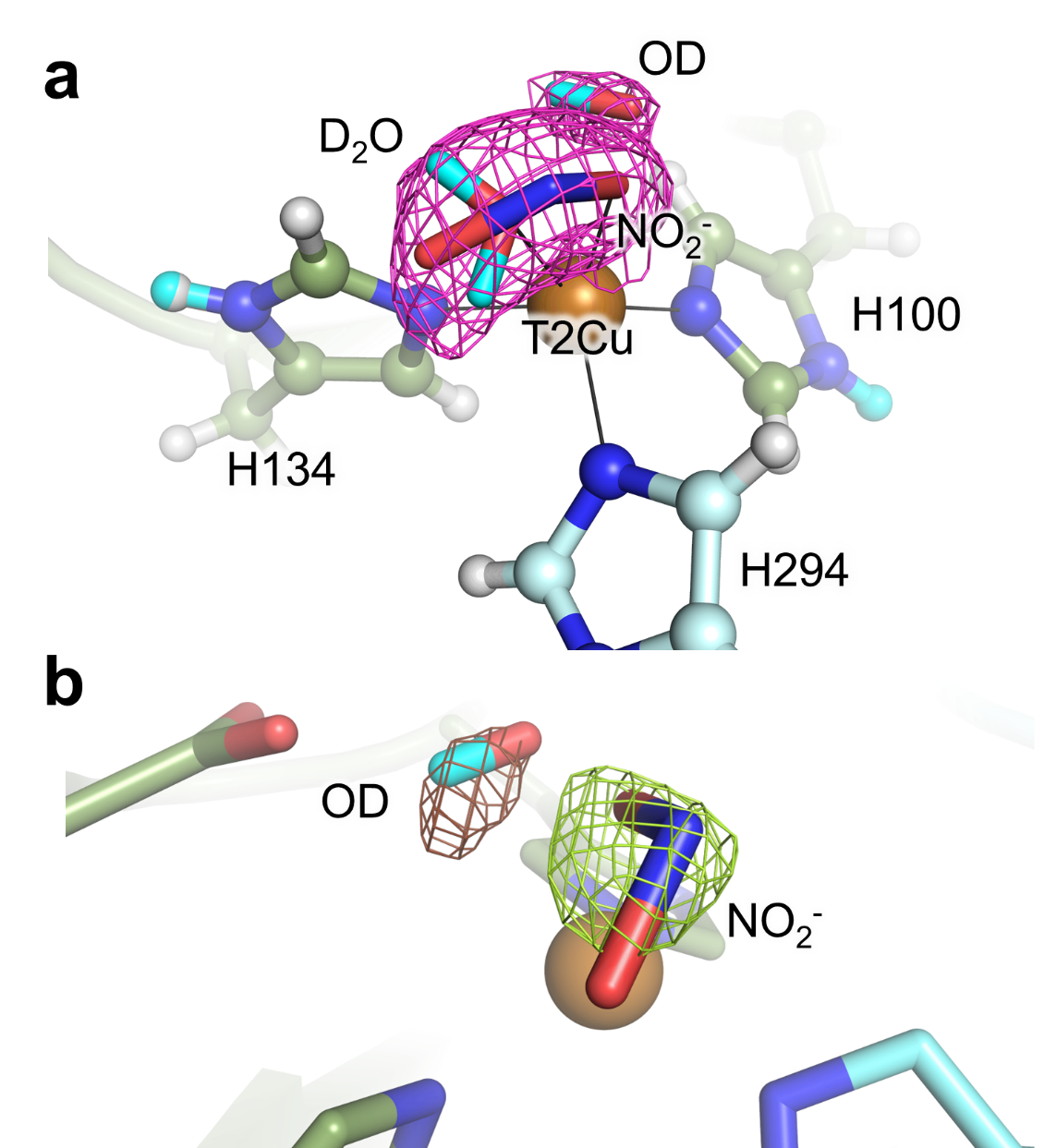
**SI Figures**

**Figure S1. Ligands at the T2Cu site in the neutron structure of the C135A-nitrite complex.** There are the major ligand nitrite (occupancy 0.7) and the minor ligand water (occupancy 0.3). OD (occupancy 0.25) is also located above the T2Cu site. The sigma-A-weighted 2*F*_o_–*F*_c_ map for neutron data is shown (magenta, contoured at 1.0σ). Black solid lines indicate coordination bonds. (b) The sigma-A-weighted *F*_o_–*F*_c_ maps for neutron data calculated by using incorrect occupancy values for NO_2_^-^ (occupancy 0.5, +3σ, green) and OD (occupancy 0.5, -2.5σ, brown), indicating unbiased data interpretation.


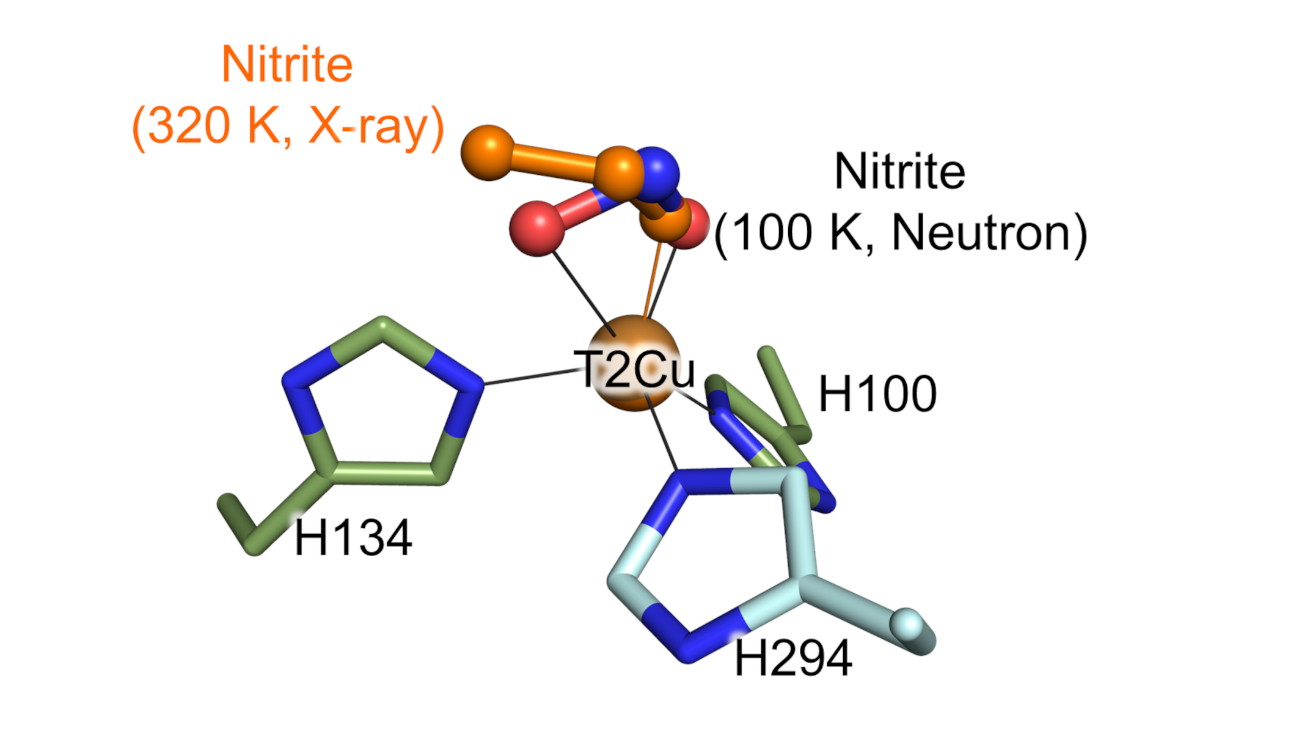


**Figure S2. Comparison between the cryogenic neutron structure and the X-ray structure determined at 320 K.** The X-ray structure at 320 K is shown by orange sticks and balls. Black and orange solid lines indicate coordination bonds. Here, nitrite observed in the neutron structure is illustrated as a κ^2^-O,O ligand for comparison.


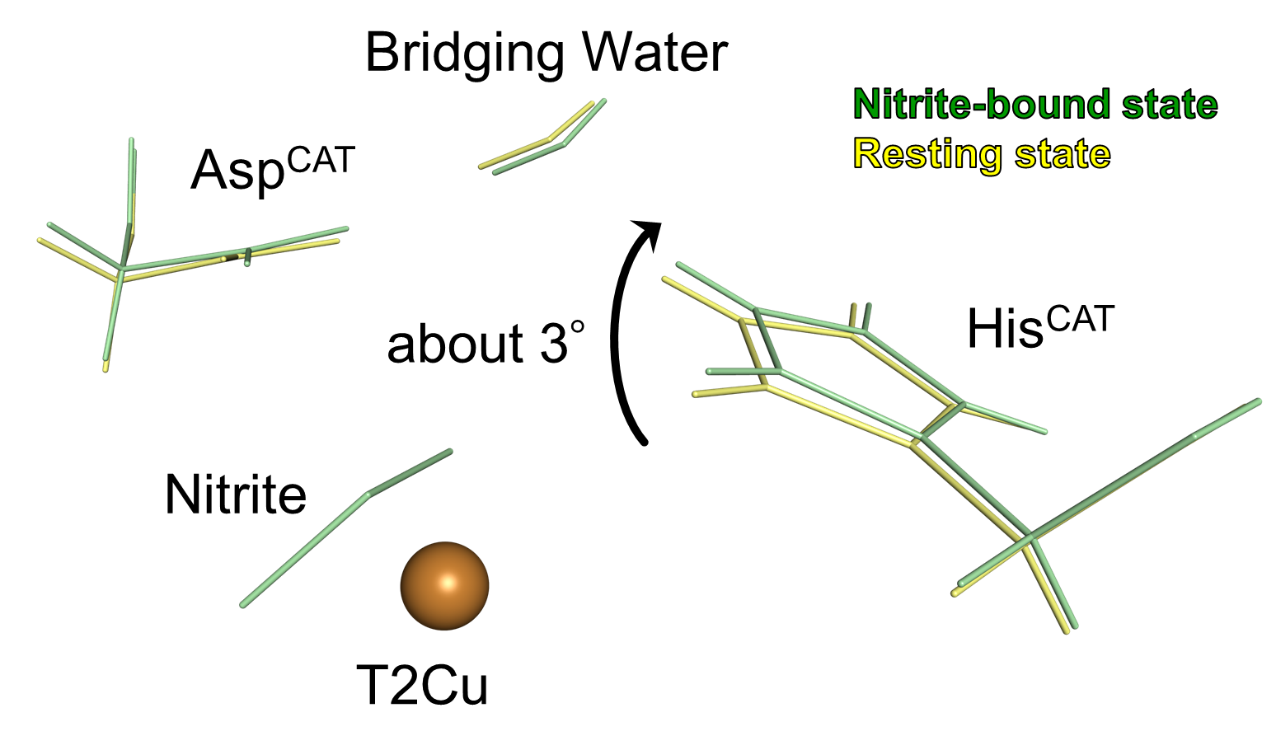


**Figure S3. Rotation of the imidazole ring of His^CAT^.** Structures are shown with sticks and balls. The neutron structure of the resting state is of PDB code ID 6L46.


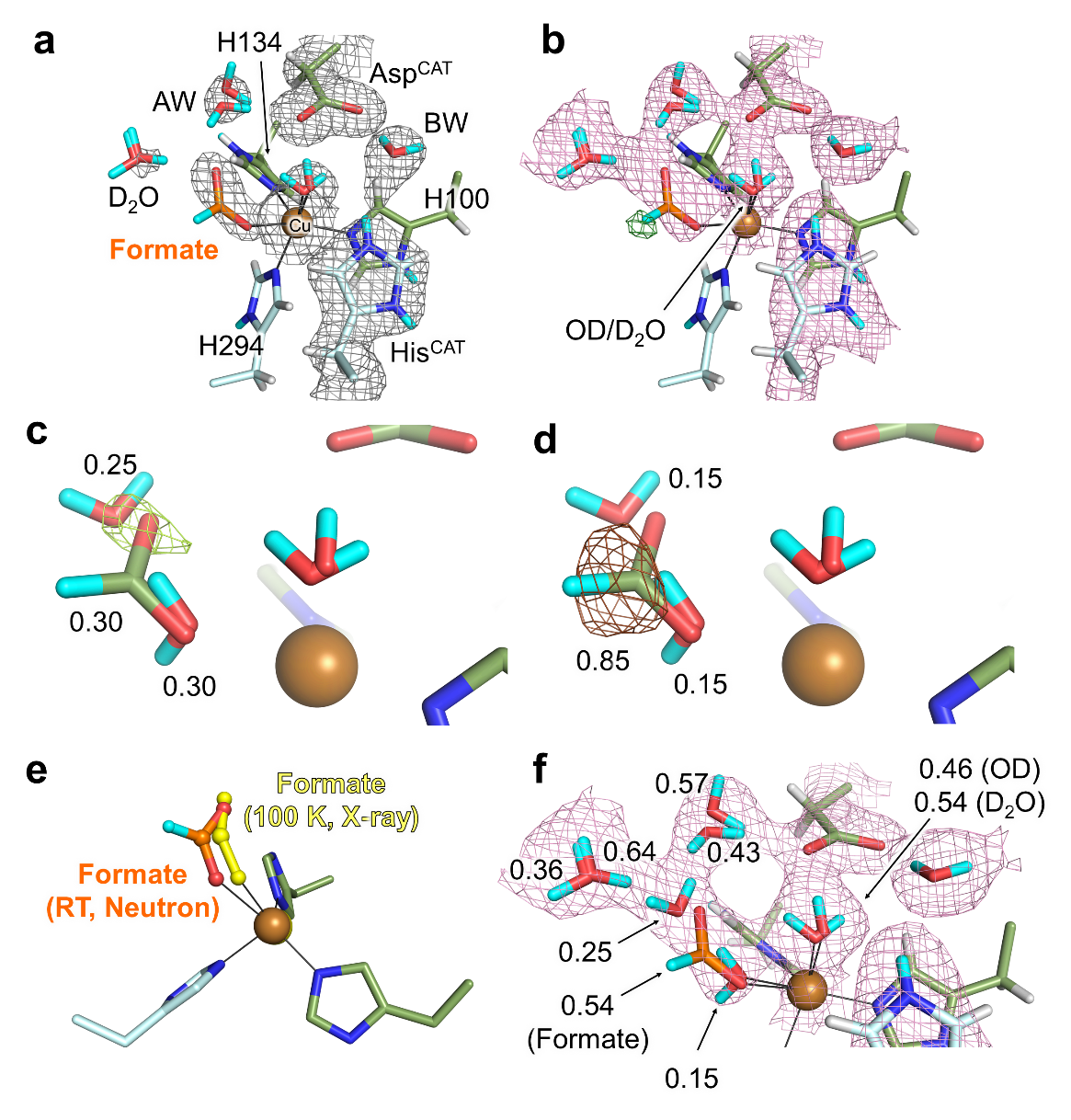


**Figure S4. Neutron structure of C135A *Gt*NIR in complex with formate.** (a) Structure around the T2Cu site. Sigma-A-weighted 2*F*_o_–*F*_c_ maps for the X-ray data contoured at 1.3σ are shown by gray meshes. Black solid lines indicate coordination bonds. For the clarity of the figure, we eliminated alternative water molecules around the formate ligand (see Fig. S3D) and a Cu ion with a very low occupancy (0.07) that located near His^CAT^ and was also observed in the previous neutron structure (PDB: 6L46) in the resting state. (b) Sigma-A-weighted 2*F*_o_–*F*_c_ maps for the neutron data contoured at 1.3σ (pink meshes). The sigma-A-weighted *F*_o_–*F*_c_ map (+ 2σ) for the neutron data, which was calculated by omitting the D atom in formate, is shown by a green mesh. (c, d) Examples of the sigma-A-weighted *F*_o_–*F*_c_ neutron maps (+ 3σ: green; - 3σ: brown) calculated by changing occupancies of molecules around the T2Cu site, indicating unbiased data interpretation. (e) Comparison of binding modes of formate between the present room temperature data and the previous cryogenic data (PDB code ID: 3WKQ). (f) Occupancies of water molecules around the T2Cu site.


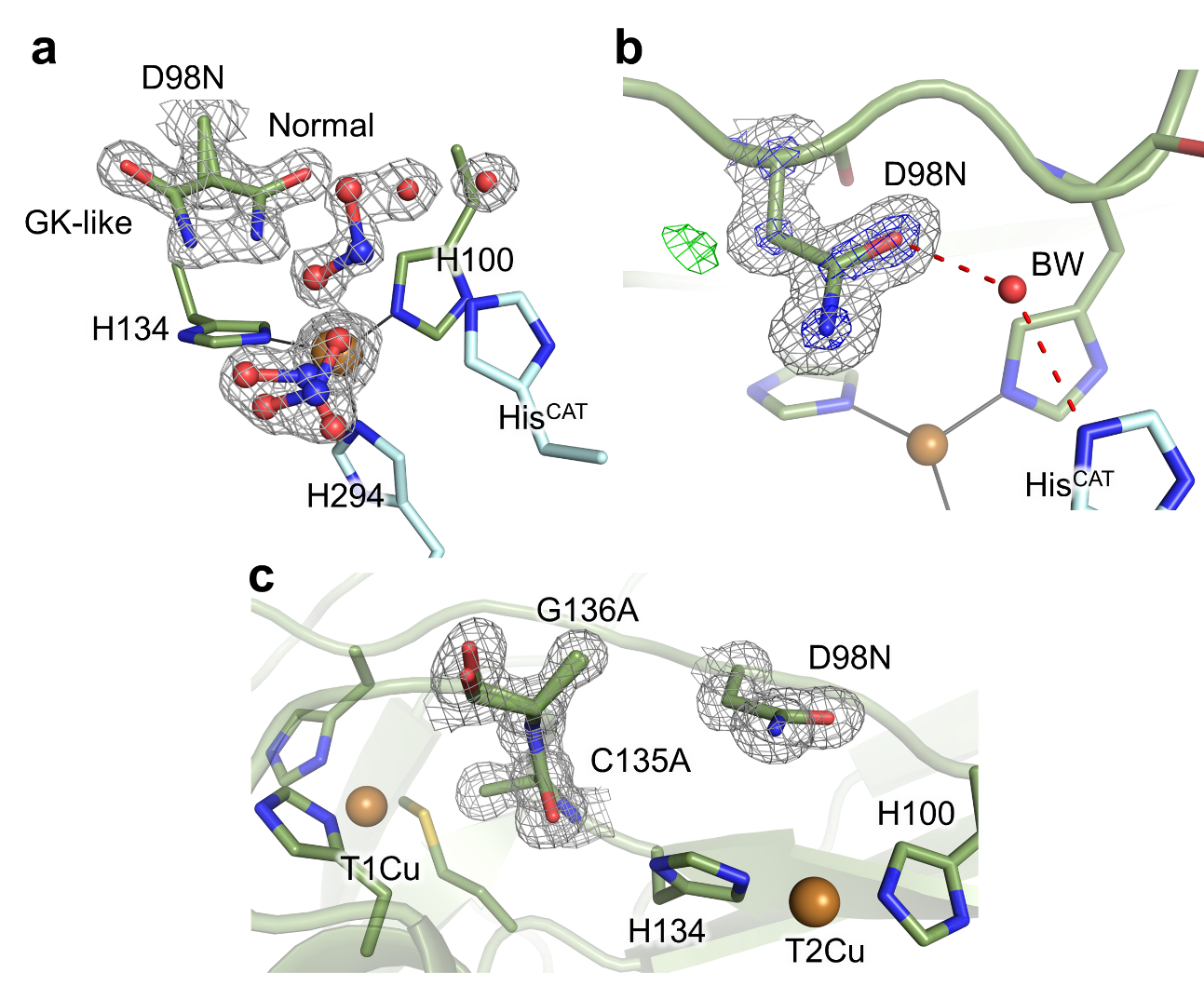


**Figure S5. Structures of D98N *Gt*NIR mutants.** (a) The structure of the D98N/C135A mutant in complex with nitrite. Sigma-A-weighted 2*F*_o_–*F*_c_ maps contoured at 1.3σ are shown by gray meshes. Black solid lines indicate coordination bonds. (b) The structure of the D98N mutant. Sigma-A-weighted 2*F*_o_–*F*_c_ maps contoured at 1 and 5σ are shown by gray and blue meshes, respectively. The sigma-A-weighted *F*_o_–*F*_c_ map contoured at 3σ is shown by a green mesh. Black solid and red dashed lines indicate coordination and H bonds, respectively. (c) Mutation positions in the D98N/C135A/G136A mutant. Sigma-A-weighted 2*F*_o_–*F*_c_ maps at 1.5σ are shown by gray mesh.


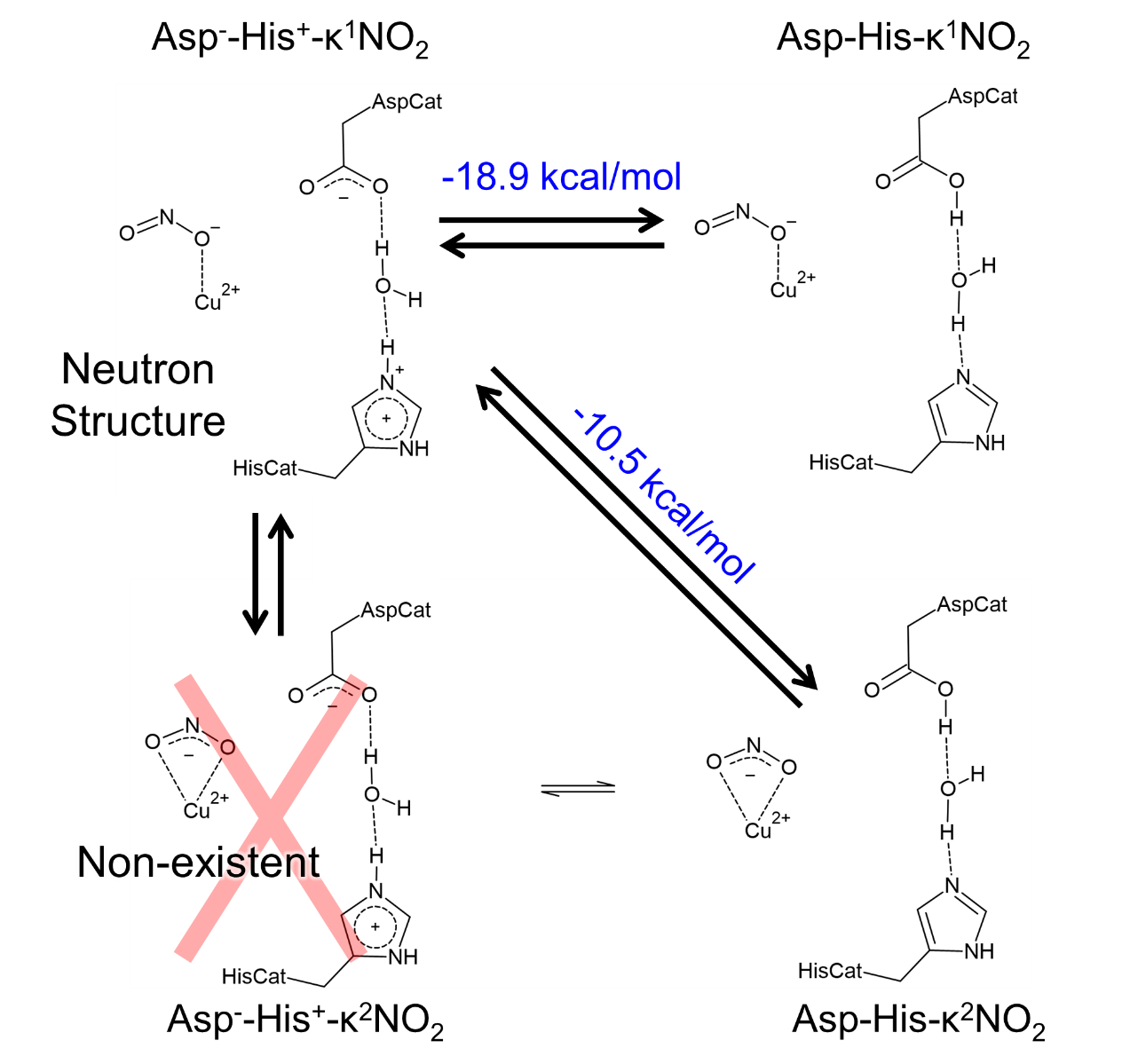


**Figure S6. Energy differences calculated by using a small QM region.**


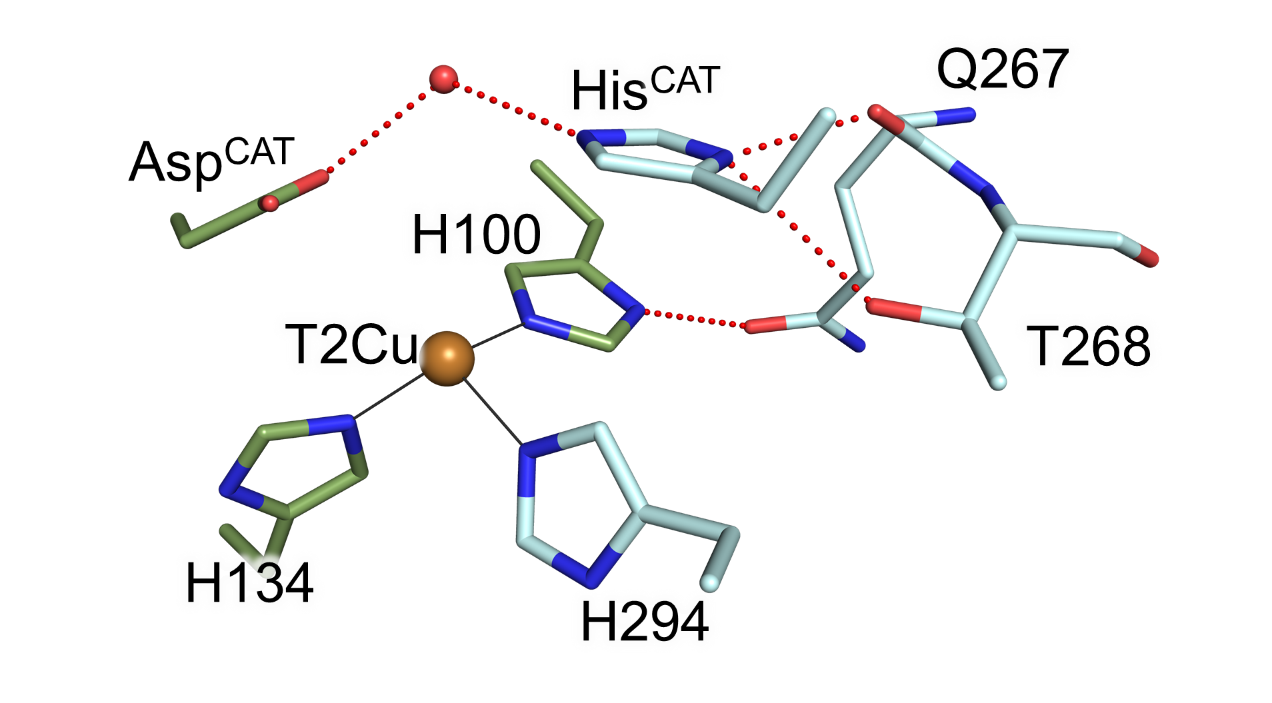


**Figure S7. Close-up view of a redox-coupled proton switch (Q267 and T268).** Coordination and hydrogen bonds are shown by black and dashed red lines.


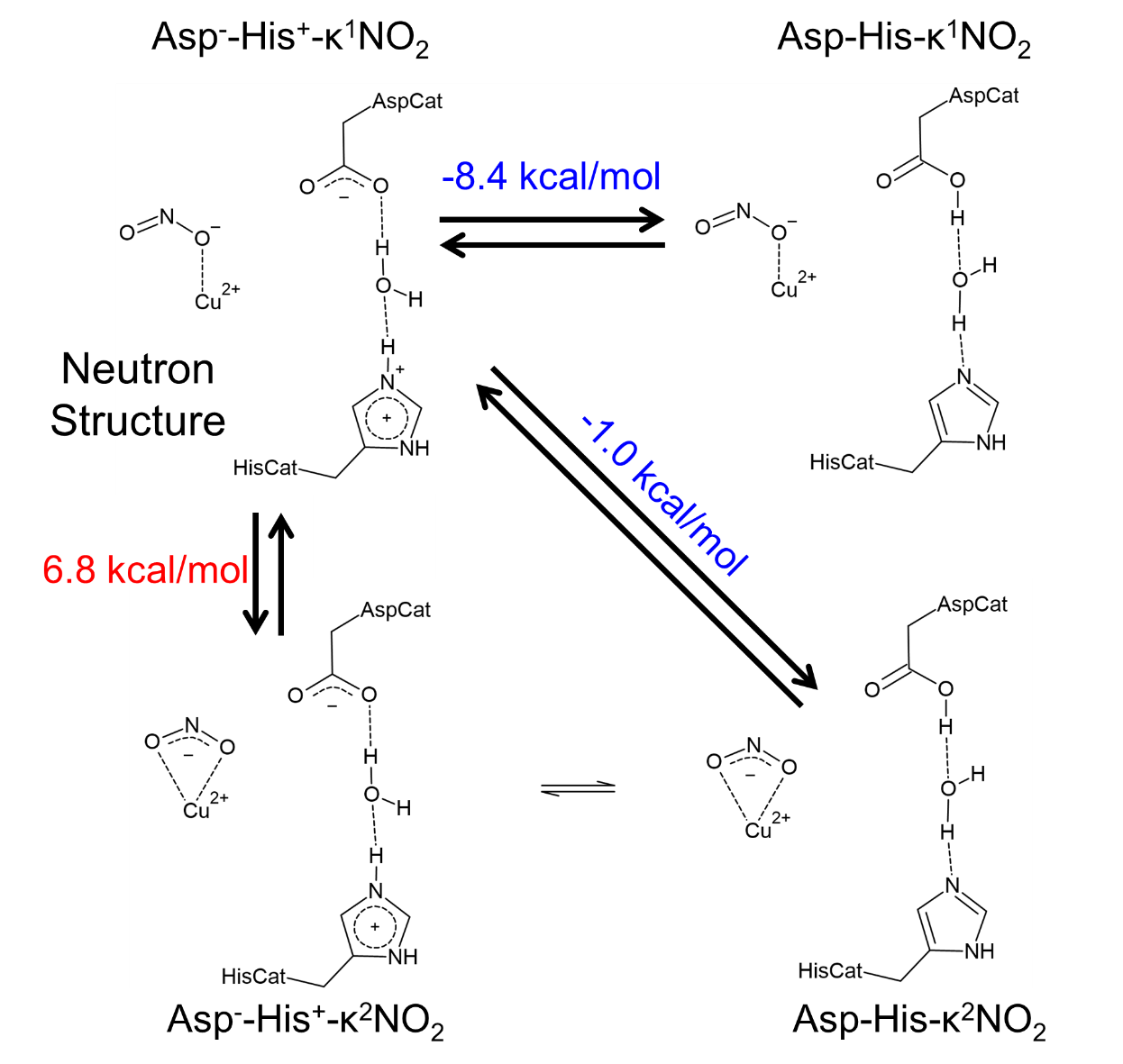


**Figure S8. Energy differences calculated by using a model including the Gln/Thr pair but without a solvent effect.**


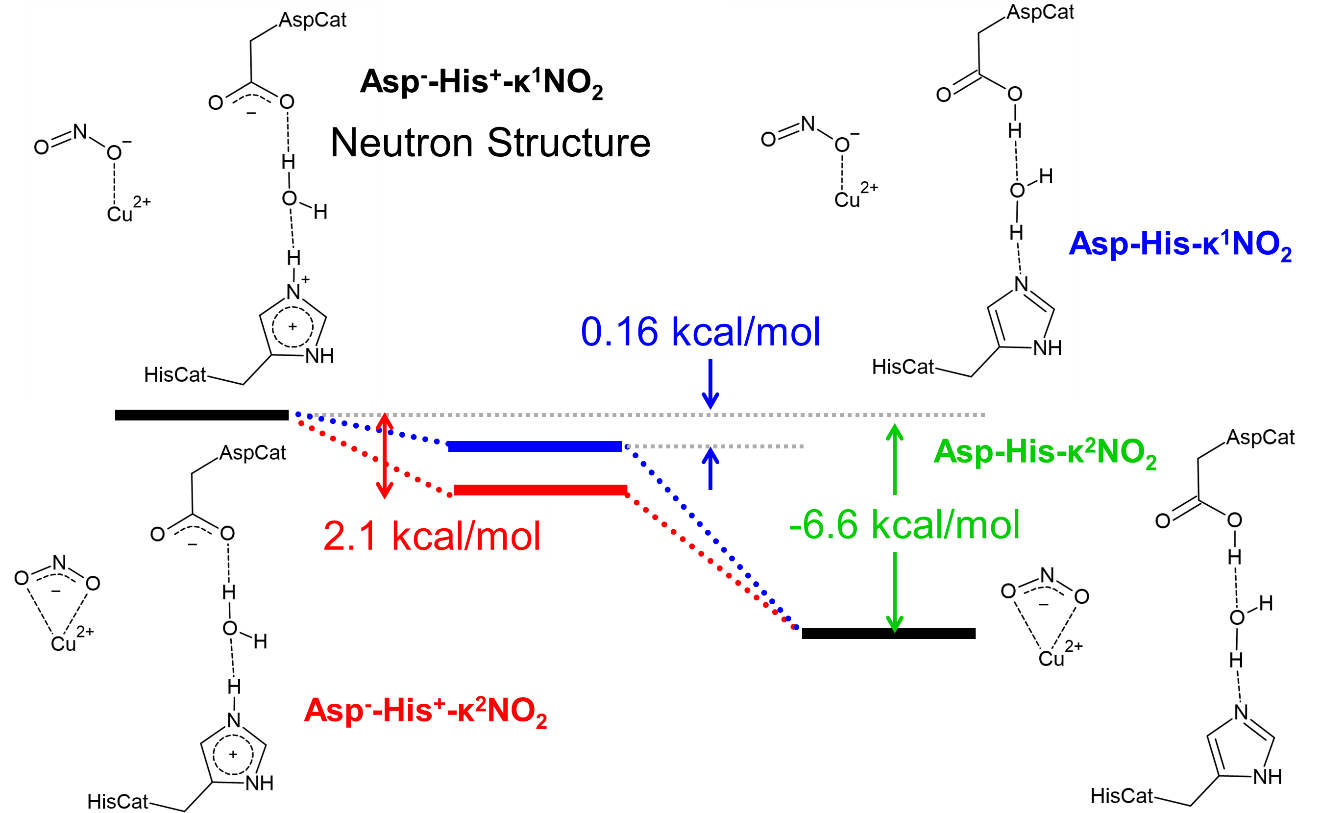


**Figure S9. Energy differences calculated by considering the Gln/Thr pair and a solvent effect at 300 K.**

**5. SI References.**

1. Y. Fukuda, Y. Hirano, K. Kusaka, T. Inoue, T. Tamada, High-resolution neutron crystallography visualizes an OH-bound resting state of a copper-containing nitrite reductase. Proc Natl Acad Sci U S A 117, 4071-4077 (2020).

2. I. Tanaka *et al*., Neutron structure analysis using the IBARAKI biological crystal diffractometer (iBIX) at J-PARC. Acta Crystallogr D Biol Crystallogr 66, 1194-1197 (2010).

3. T. Ohhara et al., Development of data processing software for a new TOF single crystal neutron diffractometer at J-PARC. Nuclear Instruments and Methods in Physics Research Section A: Accelerators, Spectrometers, Detectors and Associated Equipment 600, 195-197 (2009).

4. N. Yano et al., Status of the neutron time-of-flight single-crystal diffraction data-processing software STARGazer. Acta Crystallogr D Struct Biol 74, 1041-1052 (2018).

5. P. R. Evans, G. N. Murshudov, How good are my data and what is the resolution? Acta Crystallogr D Biol Crystallogr 69, 1204-1214 (2013).

6. W. Kabsch, Xds. Acta Crystallogr D Biol Crystallogr 66, 125-132 (2010).

7. A. Vagin, A. Teplyakov, Molecular replacement with MOLREP. Acta Crystallogr D Biol Crystallogr 66, 22-25 (2010).

8. P. V. Afonine et al., Towards automated crystallographic structure refinement with phenix.refine. Acta Crystallogr D Biol Crystallogr 68, 352-367 (2012).

9. P. Emsley, B. Lohkamp, W. G. Scott, K. Cowtan, Features and development of Coot. Acta Crystallogr D Biol Crystallogr 66, 486-501 (2010).

10. V. B. Chen et al., MolProbity: all-atom structure validation for macromolecular crystallography. Acta Crystallogr D Biol Crystallogr 66, 12-21 (2010).

11. T. Vreven et al., Combining Quantum Mechanics Methods with Molecular Mechanics Methods in ONIOM. Journal of Chemical Theory and Computation 2, 815 - 826 (2006).

12. W. D. Cornell et al., A Second Generation Force Field for the Simulation of Proteins, Nucleic Acids, and Organic Molecules. Journal of the American Chemical Society 117, 5179 - 5197 (1995).

13. M. J. Frisch et al. (2019) Gaussian 16, Revision C.02. (Gaussian, Inc., , Wallingford CT).
